# Multiple mechanisms are involved in new imazamox-resistant varieties of durum and soft wheat

**DOI:** 10.1038/s41598-017-13874-3

**Published:** 2017-11-01

**Authors:** Rafael Domínguez-Mendez, Ricardo Alcántara-de la Cruz, Antonia M. Rojano-Delgado, Pablo T. Fernández-Moreno, Raphael Aponte, Rafael De Prado

**Affiliations:** 1 Department of Agricultural Chemistry and Edaphology, University of Cordoba, 14071 Cordoba, Spain; 2 Departamento de Entomologia/BIOAGRO, Universidade Federal de Viçosa, 36570-900 Viçosa, Brazil; 3 BASF SE, Agricultural Products, 67117 Limburgerhof, Germany

## Abstract

Weed control in wheat is one of the major goals of farmers in their efforts toward obtaining the highest crop yields for human foods. Several studies (dose-response, enzyme activity, absorption-translocation and metabolism) were conducted to characterize the resistance level of two new wheat cultivars called Rafalín (*Triticum aestivum*) and Antoñín (*T. durum*) that were obtained by conventional breeding based on Clearfield® technology; they are resistant (R) to imazamox compared to their sensitive (S) counterparts (Gazul and Simeto, respectively). The R-cultivars were 93.7-fold (Rafalín) and 43.7-fold (Antoñín) more resistant than their respective S-cultivars. The acetolactate synthase (ALS) enzyme activity revealed high resistance to imidazolinone (IMI) herbicides in R-cultivars, but no cross-resistance to other ALS herbicides was found. The Ser653Asn mutation that confers resistance to IMI herbicides was identified in the *imi1* and *imi2* genes of Rafalín and only in the *imi1* gene of Antoñín. The ^14^C-imazamox absorption did not differ between the R- and S-cultivars. Imazamox was metabolized by Cyt-P450 into imazamox-hydroxyl and imazamox-glucoside in the R-cultivars, altering their translocation patterns. The differential sensitivity to imazamox between R-cultivars was due to the number of resistance genes that carry each genotype. The R-cultivars Rafalín and Antoñín could be excellent weed control tools.

## Introduction

Wheat (*Triticum* sp.) is the second most cultivated cereal in the world after maize^1^. Weeds are one of the primary biotic factors in crop production, competing for soil, water, light and nutrients^2^. In the case of wheat cultivation, they can cause a yield reduction of up to 50%^3^.

Since the emergence of 2,4-dichlorophenoxyacetic acid (2,4-D) in the mid-1940s^4^, weed control in wheat has improved thanks to the availability of a variety of selective active ingredients belonging to different chemical families, among which the following should be mentioned: 2,4-D, MCPA, MCPP, bentazon, bromoxynil, ioxynil, diclofop, fenoxaprop, clodinafop, iodosulfuron, mesosulfuron, pinoxaden, etc. Another weed control modality in crops is the use of non-selective herbicides (broad spectrum), for which it is necessary to choose wheat varieties that are resistant to the active herbicide ingredients^5,6^.

In recent years, some crop varieties with resistance to imidazolinone (IMI) herbicides [acetolactate synthase (ALS, EC 2.2.1.6; also known as acetohydroxyacid synthase: AHAS) inhibitors group] have been developed. These herbicides are classified as broad-spectrum weed control. These crops are known as “IMI varieties,” and their development primarily involves Clearfield® technology^7^. IMI-resistant crops can be grown from two to a maximum of four years in the same field to reduce the risk of developing herbicide-resistant weeds^8^.

In most cases, resistance to ALS-inhibiting herbicides is due to a mutation at the site of action, although there are some cases in which the responsible mechanism is a rapid detoxification of the herbicide by the plant’s metabolism^9–11^. Regarding exchanges in the ALS gene that confer resistance to ALS-inhibiting herbicides, eight have been described, and they result in exchanges in the amino acid positions Ala122, Pro197, Ala205, Asp376, Arg377, Trp574, Ser653 and Ser654^10^. The Trp574Gly mutation confers cross-resistance to the entire family of ALS-inhibiting herbicides, while the mutations in the Pro197Ser or Pro197Ala codons are more resistant to the sulfonylurea family. The exchanges Ala122Thr, Ala205Val, Ser653Asn and Ser653Thr confer resistance to IMI; Ala122Thr confers even higher levels of resistance to IMI than exchanges involving Ser653^12^. In IMI-resistant crops, the increase in resistance is a consequence of having two or more resistant genes in a single genotype^13^.

Durum wheat (*Triticum durum*) is tetraploid (28 chromosomes), while soft wheat (*T. aestivum*) is hexaploid (42 chromosomes)^14^. Three homologous genes of ALS in wheat have been identified, and they are known as *imi1, imi2* and *imi3* (also known as *ahas*L-B1, *ahas*L-D1 and *ahas*L-A1, respectively). These genes are located in chromosomes 6B, 6D, and 6A, respectively^13,15^.

Wheat varieties that are resistant to herbicides are an attractive alternative for weed control^16^, and they improve production. Several studies have demonstrated that GM crops do not have side effects on non-target organisms^17,18^. However, EU bureaucracy and adoption challenges may lead to an uphill struggle for marketing GM wheat varieties^19^. Obtaining new herbicide-resistant wheat varieties by conventional plant breeding could facilitate their implementation and acceptance by the EU bureaucracy.

The aim of this work was to determine the IMI herbicide resistance levels of two new wheat cultivars (soft var. Rafalín and durum var. Antoñín) that were obtained by conventional crossbreeding with susceptible Spanish cultivars and to characterize the relevant resistance mechanisms.

## Results

### Foliar retention

The mean imazamox solution amounts retained on the leaves of the different wheat plants were 103 ± 2.1, 92 ± 7.9, 96 ± 4.5 and 90 ± 8.1 μL g^−1^ dry weight in Gazul, Rafalín, Simeto and Antoñín, respectively, with no differences between them.

### Dose-response and ALS activity tests

**Figure 1:**
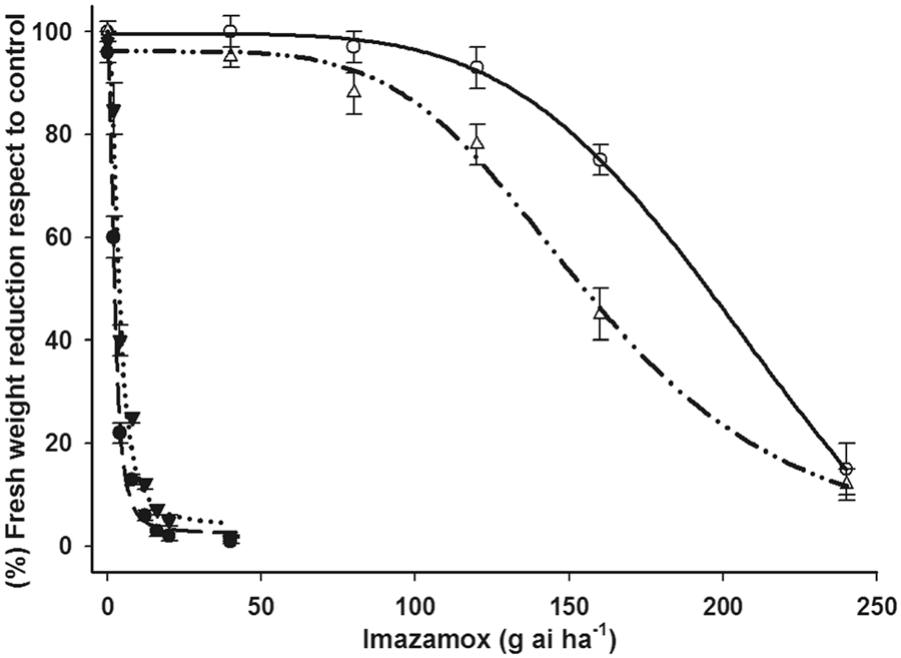
Dose-response curves of the fresh weight reduction with respect to untreated control plants of imazamox-susceptible and imazamox-resistant cultivars from the different soft (*Triticum aestivum*) and durum (*Triticum durum*) wheat varieties when evaluated at 30 DAA. Vertical bars ± standard error (n = 10).

Imazamox resistance was confirmed in the two new Rafalín and Antoñín cultivars. Data on the fresh plant weights fit well to the log-logistic non-linear regression model, allowing for the estimation of effective mean doses that reduced the fresh weight by 50% (GR_50_). Soft wheat cultivars presented GR_50_ values of 2.4 and 224.8 g ai ha^−1^ for imazamox in Gazul and Rafalín, respectively. In addition, the GR_50_ of the durum wheat cultivars were 3.6 and 157.2 g ai ha^−1^ for Simeto and Antoñín, respectively. The resistance factors (RF) of the R-cultivars were 43.7 and 93.7 for Antoñín and Rafalín, respectively, relative to their corresponding S-cultivars (Fig. 1, Table 1).

**Table 1:** Parameters of the sigmoidal equation used to estimate the imazamox dose (g ai ha^−1^) needed to reduce the weight of a population by 50% (GR_50_) in susceptible and resistant wheat cultivars of the soft (*Triticum aestivum*) and durum (*Triticum durum*) varieties.

Variety	Cultivar	c	d	b	R^2^ aj	*P-*value	GR_50_ (CI95)	RF
Soft	Rafalín (R)	99.5	−0.6	4.7	0.99	<0.0001	224.8 (17.6)	93.7
	Gazul (S)	96.2	2.4	2.26	0.99	0.0011	2.4 (0.6)	—
Durum	Antoñín (R)	96.2	0.4	4.7	0.98	0.0176	157.2 (12.9)	43.7
	Simeto (S)	99.9	4.1	2.2	0.97	0.0072	3.6 (1.1)	—

c = lower limit, d = upper limit, b = Hill’s slope, R^2^ aj = 1 − (sums of squares of the regression/corrected total sums of squares). RF = Resistance factor = GR_50_R/GR_50_S. CI95 values are the upper and lower limits (±) of the 95% confidence intervals (*n* = 10).

**Figure 2:**
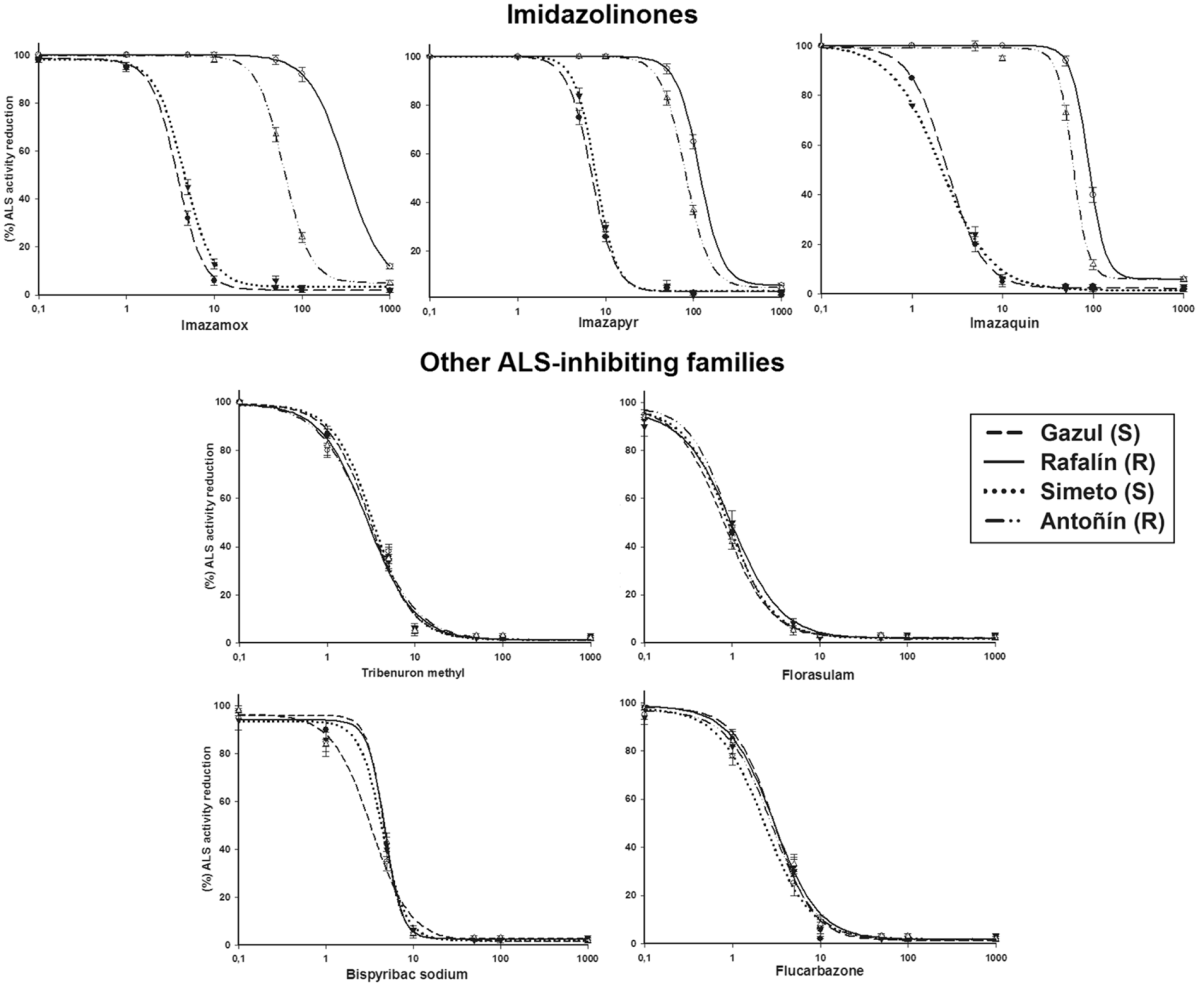
Log-logistic curves of five ALS inhibitor families on the ALS activity in imazamox-susceptible and imazamox-resistant plants of the soft (*Triticum aestivum*) and durum (*Triticum durum*) wheat varieties. Vertical bars ± standard error (n = 3).

The specific *in vitro* activities of the ALS enzyme in Gazul, Rafalín, Simeto and Antoñín were 287, 276, 302 and 293 nmol acetoin mg^−1^ protein h^−1^, respectively, with no significant differences. The imazamox inhibited the ALS activity in all the cultivars as the concentrations increased. To inhibit the ALS activity by 50% (I_50_), 3.8 and 5.3 µM imazamox were required for Gazul and Simeto (S-cultivars), respectively. The R-cultivars Antoñín and Rafalín presented RFs that were 13.8 and 82.7 times higher, respectively, relative to their corresponding S-cultivar. The R-cultivars showed multiple resistance to herbicides in the IMI family; however, they were not resistant to the other families of ALS inhibitor herbicides (Fig. 2, Table 2).

**Table 2:** Parameters of the sigmoidal equation used to estimate the concentration (µM) of ALS-inhibiting herbicides needed to inhibit the ALS activity by 50% (I_50_) in imazamox-susceptible and imazamox-resistant soft (*Triticum aestivum*) and durum (*Triticum durum*) wheat varieties.

Herbicide	Cultivar	c	d	b	R^2^ aj	*P-*value	I_50_ (CI95)	RF^b^
Imidazolinones								
Imazamox	Rafalín	100.0	4.2	2.1	1.00	<0.0001	314.1 (23.7)	82.7
	Gazul	98.3	2.0	2.9	0.99	<0.0001	3.8 (0.93)	—
	Antoñín	99.7	4.9	2.9	0.99	<0.0001	62.3 (8.2)	13.8
	Simeto	98.3	3.4	2.6	0.99	<0.0001	4.5 (0.87)	—
Imazapyr	Rafalín	100.0	5.4	3.4	0.99	<0.0001	176.6 (14.5)	25.6
	Gazul	100.0	3.2	3.2	0.99	<0.0001	6.9 (0.64)	—
	Antoñín	100.0	4.9	3.1	0.99	<0.0001	80.7 (6.2)	10.6
	Simeto	100.0	3.6	3.7	0.99	<0.0001	7.6 (0.81)	—
Imazaquin	Rafalín	100.0	5.9	4.6	1.00	<0.0001	88.6 (5.7)	36.9
	Gazul	99.9	2.3	2.1	0.99	0.0002	2.4 (0.23)	—
	Antoñín	99.0	5.9	5.2	0.99	<0.0001	59.9 (4.3)	28.5
	Simeto	100.2	1.5	1.6	0.99	<0.0001	2.1 (0.16)	—
Other ALS-inhibiting families								
Tribenuron methyl (SU)	Rafalín	99.4	0.9	1.5	0.98	<0.0001	2.4 (0.11)	0.8
	Gazul	100.0	1.8	1.1	0.96	0.0066	2.9 (0.22)	—
	Antoñín	99.7	0.8	1.3	0.99	<0.0001	2.7 (0.31)	0.9
	Simeto	100.0	1.1	1.1	0.98	<0.0001	2.8 (0.14)	—
Florasulam (TP)	Rafalín	100.1	2.0	1.1	0.96	<0.0001	1.3 (0.23)	1.4
	Gazul	96.5	1.7	1.6	0.99	<0.0001	0.9 (0.14)	—
	Antoñín	100.0	2.1	1.3	0.98	0.0005	1.2 (0.17)	1.5
	Simeto	99.7	2.3	1.1	0.99	<0.0001	0.8 (0.11)	—
Bispyribac sodium (PTB)	Rafalín	96.0	2.5	4.5	0.99	<0.0001	4.7 (0.86)	1.1
	Gazul	93.6	1.9	3.3	0.98	0.0011	4.4 (0.41)	—
	Antoñín	94.0	2.4	4.4	0.98	0.0176	4.7 (0.62)	1.4
	Simeto	96.4	1.4	1.9	0.98	0.0072	3.3 (0.75)	—
Flucarbazone (SCT)	Rafalín	97.6	1.8	1.6	0.99	0.0004	2.3 (0.20)	0.8
	Gazul	98.1	1.1	1.9	0.98	0.0028	3.0 (0.45)	—
	Antoñín	96.7	1.3	1.7	0.99	0.0010	2.8 (0.32)	0.9
	Simeto	98.6	1.6	1.7	0.99	0.0004	2.9 (0.27)	—

c = lower limit, d = upper limit, b = Hill’s slope, R^2^ aj = 1 − (sums of squares of the regression/corrected total sums of squares). RF = Resistance factor = I_50_R/I_50_S. ^c^CI values are the 95% confidence intervals (n = 3). SU = Sulfonylureas. PTB = Pyrimidinylthiobenzoates. SCT = Sulfonylaminocarbonyltriazolinone. TP = Triazolopyrimidines.

According to the 95% confidence intervals (CI), the S-cultivars showed no significant differences in either the GR_50_ or I_50_ parameters. Independent of the wheat variety, the Rafalín cultivar is the one that withstands a higher dose of imazamox.

### ALS sequencing

**Figure 3:**
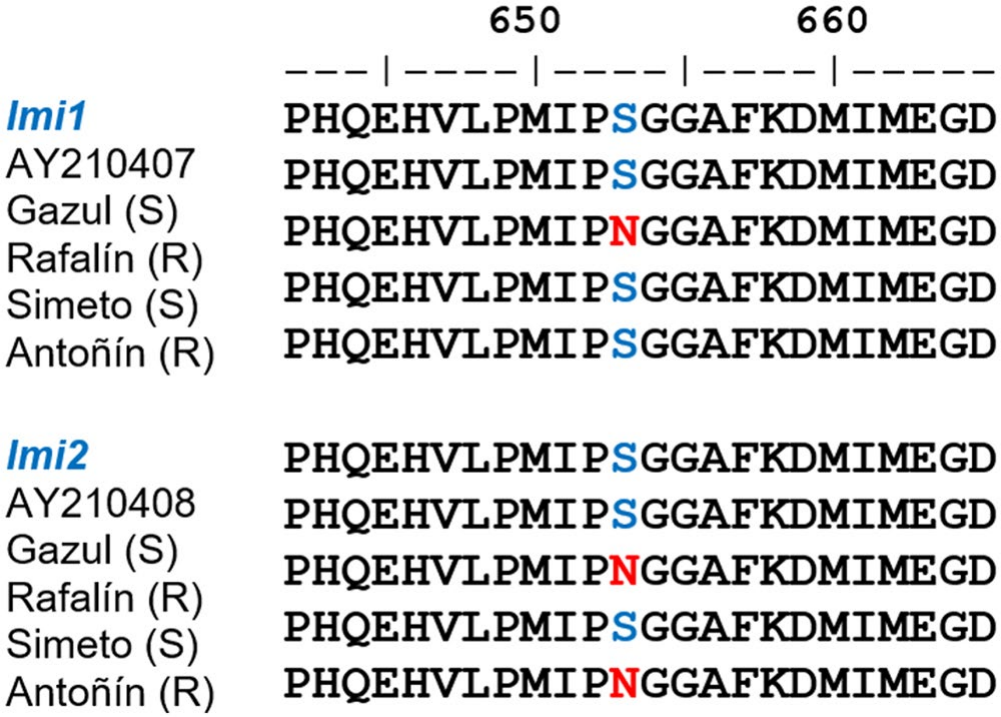
Partial alignment of amino acid sequences for the *imi1*-ALS and *imi2*-ALS genes of the imazamox-susceptible and imazamox-resistant cultivars of the different soft (*Triticum aestivum*) and durum (*Triticum durum*) wheat varieties. Colored letters indicate the Ser-653 position corresponding to the point mutation associated with the conferring of imazamox resistance. Red letters indicate a change at the 653 position from AAC (serine = S) to AGC (asparagine = N) in the consensus nucleotide sequence.

The predicted amino acid sequence of S cultivars was presented as the same consensus of accessions *imi1*-AY210407 and *imi2*-AY210408 in wheat, corresponding to the *imi1* and *imi2* genes, respectively. The R-cultivar Rafalín presented two mutations at the Ser653 position (also known as Ser627^15,20^) in the *imi1* and *imi2* genes, whereas the Antoñín cultivar presented the same mutation, but in the *imi2* gene. The codon change was AAC to AGC, resulting in an amino acid substitution from serine to asparagine. No mutation was found in the *imi3* gene (Fig. 3).

### ^14^C-Imazamox absorption and translocation

**Figure 4:**
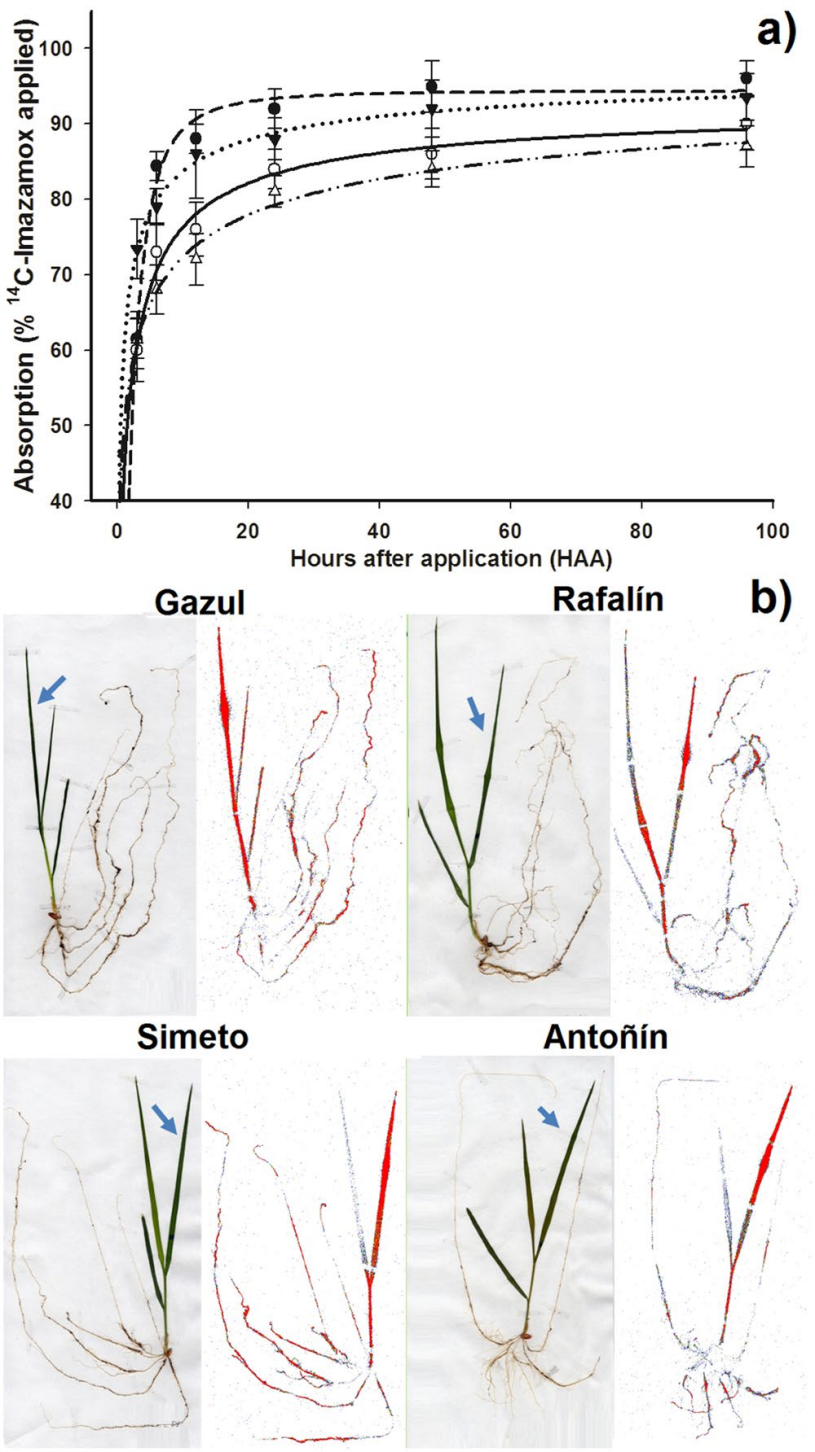
^14^C-imazamox absorption and translocation in wheat imazamox-susceptible and imazamox-resistant cultivars of the different soft (*Triticum aestivum*) and durum (*Triticum durum*) wheat varieties. (**a**) ^14^C-imazamox absorption in the imazamox-susceptible and imazamox-resistant wheat plants. Vertical bars ± standard error (n = 5). (**b**) Digital images and autoradiograph images of ^14^C-imazamox translocation in imazamox-susceptible and imazamox-resistant wheat plants at 96 HAA. The highest concentration of ^14^C-imazamox is highlighted in red. Arrows indicate the treated leaf.

The four wheat cultivars presented a high ^14^C-imazamox absorption rate. At 12 h after application (HAA), the S-cultivars presented an absorption level of over 87%, absorbing up to more than 94% at 96 HAA, while the R cultivars exhibited an average absorption of 73 and 88% at 12 and 96 HAA, respectively. Although the S-cultivars absorbed more ^14^C-imazamox, the differences were not significant with respect to the R-cultivars (Fig. 4a).

The S-cultivars showed high rates of ^14^C-imazamox translocation from the treated leaves to the rest of the plants and roots. The greatest differences in translocation were observed at 96 HAA. The rates of translocation to the roots of the R-cultivars were 16.1 and 16.6% for Rafalín and Antoñín, respectively, for the absorbed herbicide, translocating 8.5–10.3% less herbicide to the roots than the S-cultivars and retaining approximately 60% in the treated leaves of both imazamox-resistant cultivars. At that time, the S-cultivars presented a higher translocation of ^14^C-imazamox from the treated leaves to the rest of the plants and roots (Table 3).

**Table 3:** Translocation percentage of ^14^C-imazamox in imazamox-susceptible and imazamox-resistant wheat plants of the durum (*Triticum durum*) and soft (*Triticum aestivum*) varieties.

Cultivar	HAA^a^	Imazamox translocation (% from absorbed)		
		Treated leaf	Rest of shoots	Root
***Wheat soft varieties***				
Gazul (S)	3	95.1 ± 1.1 a	2.2 ± 0.7 h	2.6 ± 1.3 g
	6	88.8 ± 2.8 b	3.3 ± 2.5 h	7.9 ± 0.8 f
	12	73.3 ± 2.5 d	12.8 ± 2.3 f	13.9 ± 0.9 e
	24	65.6 ± 1.5 ef	14.5 ± 1.9 e	19.9 ± 1.8 c
	48	54.3 ± 3.9 g	22.9 ± 3.0 b	22.8 ± 2.1 b
	96	45.4 ± 2.5 h	30.0 ± 1.7 a	24.6 ± 1.8 a
Rafalín (R)	3	94.2 ± 1.5 a	3.3 ± 0.4 h	2.6 ± 1.1 g
	6	88.8 ± 3.3 b	2.9 ± 2.3 h	8.3 ± 1.2 f
	12	79.7 ± 3.3 c	6.9 ± 1.1 g	13.3 ± 2.6 e
	24	70.2 ± 2.4 de	16.1 ± 1.3 d	13.8 ± 1.9 e
	48	67.3 ± 4.1 e	18.6 ± 2.8 c	14.2 ± 3.0 e
	96	61.9 ± 3.6 f	22.1 ± 1.4 b	16.1 ± 2.0 d
***Wheat durum varieties***				
Simeto (S)	3	92.4 ± 1.0 A	3.6 ± 0.6 H	4.0 ± 0.5 H
	6	84.8 ± 2.9 B	9.6 ± 3.1 G	5.6 ± 0.5 G
	12	77.1 ± 1.5 C	9.0 ± 1.3 G	13.9 ± 0.3 D
	24	51.7 ± 0.7 G	20.9 ± 1.9 D	27.3 ± 2.4 A
	48	42.6 ± 1.2 H	31.8 ± 2.1 B	25.6 ± 2.2 B
	96	38.0 ± 4.6 I	35.1 ± 1.7 A	26.9 ± 3.0 AB
Antoñín (R)	3	94.5 ± 1.7 A	2.2 ± 0.1 H	3.3 ± 1.6 H
	6	83.4 ± 1.5 B	8.3 ± 0.7 G	8.3 ± 1.2 F
	12	73.3 ± 3.1 C	15.5 ± 4.7 F	11.2 ± 1.6 E
	24	69.1 ± 3.1 D	18.5 ± 1.4 E	12.4 ± 2.1 DE
	48	63.9 ± 3.0 E	22.3 ± 2.4 D	13.9 ± 2.8 D
	96	59.2 ± 1.4 F	19.2 ± 1.7 C	16.6 ± 2.1 C

^a^HAA: Hours after application. Means with different letter within a column are statistically different at 95% probability determined by the Tukey’s test. ±Standard error of the mean (n = 5).

A Phosphor Imager was used to confirm the previous results. At 96 HAA, the R-cultivar plants translocated smaller amounts of ^14^C-imazamox from the treated leaf to the roots than S-cultivar plants. This finding shows that the ^14^C-imazamox translocation could have contributed to the resistance of the R-cultivars (Fig. 4b).

### Imazamox metabolism

**Figure 5:**
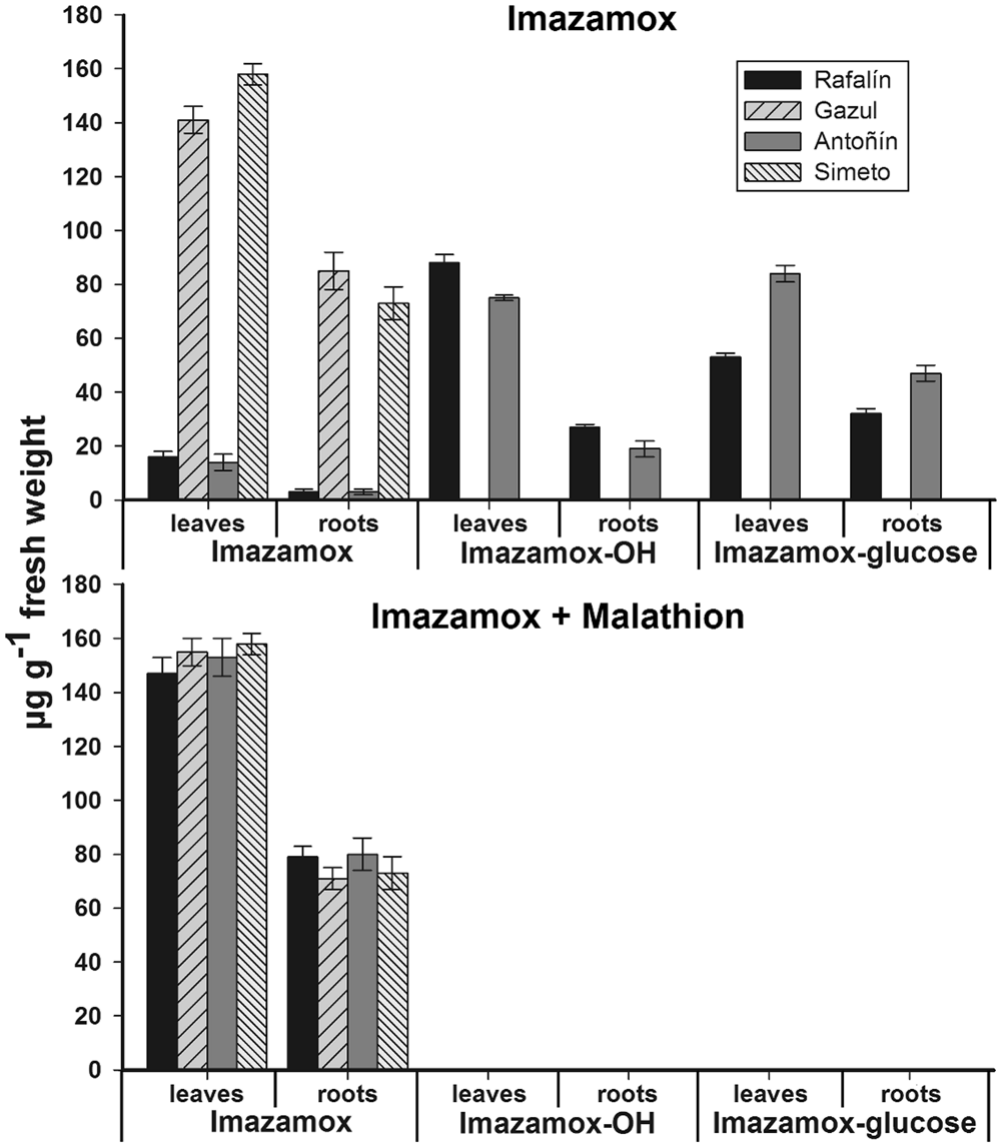
Imazamox metabolism of soft (*Triticum aestivum*) and durum (*Triticum durum*) wheat varieties treated at the field dose (40 g ai ha^−1^ imazamox). A) The total concentration of imazamox and its metabolites in leaf and root samples from imazamox-susceptible and imazamox-resistant cultivars of the different wheat varieties at 96 HAA as obtained by LC-DAD and LC-TOF/MS. Bars indicate the standard deviation of the mean (n = 3).

In this study, the R-cultivar plants (Rafalín and Antoñín) presented rapid imazamox metabolism to hydroxylated imazamox (imazamox-OH) and a glucose conjugate (imazamox-glucose), which were metabolites that were not found in the S-cultivars (Gazul and Simeto). In taking into account that these metabolites come from imazamox, we considered the sum of all of them to be 100%, and we calculated the imazamox percentage that was metabolized in the leaves and roots. The R-cultivars had a high metabolite content at 96 HAA compared to the S-cultivars. In the case of the Rafalín and Antoñín cultivars, over 90% corresponded to imazamox metabolites, while the Gazul and Simeto cultivars did not metabolize this herbicide. It should be noted that the Antoñín cultivar presented higher contents of the glycosylated metabolite (84.1 μg g^−1^ in leaves and 47.6 μg g^−1^ in roots) compared to the Rafalín cultivar (50.4 μg g^−1^ in the leaves and 29.1 μg g^−1^ in the roots). Imazamox metabolism inhibition was also observed in R-cultivar plants that were treated with malathion. This finding suggests that cytochrome P450 monooxygenase (Cyt-P450) is involved in this detoxification mechanism (Fig. 5).

## Discussion

Clearfield® wheat that is resistant to imazamox is a new and highly effective tool for weed control that has been developed in North America and in most Latin American countries. This study reports the first European case of two imazamox-resistant wheat cultivars, based on Clearfield® technology, which were obtained by the crossbreeding of Pantera *T. aestivum* Clearfield®^20^ and a local imazamox-susceptible cultivar (Gazul) and another *Triticum durum* Clearfield® obtained by the crossing of Simeto (local imazamox-susceptible *T. durum* cultivar) and a *Tritordeum* Clearfield®^15^.

Dose-response assays demonstrated the high susceptibility of the S-cultivars (Gazul and Simeto) at low doses, whereas the R-cultivars (Rafalín and Antoñín) presented a small reduction in fresh weight. The differences between the R-cultivars could be explained by the different biochemical, morphological, physiological and molecular traits^21–23^. However, to achieve total growth reduction in a resistant plant, the grower needs to apply at least double the rate of herbicide as that of its corresponding GR_50_^24^. This rule implies that the Antoñín and Rafalín cultivars require imazamox doses that are 8 and 11 times, respectively, higher than the recommended field dose of 40 g ia ha^−1^ of imazamox to produce total damage in the crop. This high level of resistance to the imazamox of wheat R-cultivars is enough to be a useful weed control tool, with advantages for farmers. The adequate use of herbicide-tolerant crops and the adoption of the associated agronomic practices may enhance farmland biodiversity and reduce the risk of weeds evolving herbicide resistance^8^.

Resistance to IMI herbicides is usually the result of a point mutation in the ALS gene that causes an amino acid substitution in the ALS enzyme^10,25^. The high I_50_ rate of the R-cultivars suggests that the resistance mechanism is related to the ALS enzyme in wheat cultivars^9,16,20^. The similar specific activity of ALS between them also suggests that ALS overexpression is not involved as a resistance mechanism. Similar results were described in *Sinapis alba* ^26^ and *T. aestivum* ^27^, in which ALS overexpression was not involved as a resistance mechanism.

The higher GR_50_ and I_50_ values estimated for Rafalín were due to the fact that this cultivar presented a mutation at the Ser-653 position in the *imi1* and *imi2* genes, which are located on the long arms of chromosomes 6B and 6D, respectively. Consequently, this resistant cultivar has a higher imazamox resistance level than the Antoñin cultivar, and it only carried a single resistance gene (*imi2*). The R-wheat cultivars have satisfactorily acquired these alleles from their respective resistant parents, i.e., the *imi1* and *imi2* genes from the Pantera^20^ cultivar were transferred to Rafalín, and *imi2* from *Tritordeum* ^15^ was transferred to the Antoñín cultivar. The resistant allele *imi-2*, common in Clearfield® crops, endows sufficient resistance level at recommended field rates to IMI herbicides^28^. The resistance conferred by the ALS-resistant *imi1* gene resulted in an additive resistance level to that conferred by *imi2* gene. These mutations only confer resistance to IMI herbicides, but not cross-resistance to other ALS inhibitors^6^. The fitness cost associated to ALS-resistant alleles is small and easily detectable^10^. However, this fact can not generalize and the impact of each specific ALS gene mutation needs to be individually evaluated^29^, taking into account the growing conditions and species^10^. In addition, the epistatic effects of multiple resistance alleles on plant fitness cost is yet unknown^10,30^. Therefore, further studies are needed to determine the epistatic effect of ALS-resistant allele in *imi1* gene of cultivar Rafalín, on fitness cost and the possible yield drag.

The ALS isoform from the D genome, which corresponds to the *imi2* gene, presents more ALS activity compared to isoforms of the A and B genomes^31^. This finding explains why the *imi2* mutation is sufficient for making the Antoñín cultivar resistant to imazamox. Our results are consistent with other studies that report a single and/or double mutation in the *imi1-* and *imi2*-ALS genes that confer resistance to IMI herbicides in wheat cultivars^16,20,28,32^, and other Clearfield^®^ crops such as rice^33^, barley^34^, sunflowers^35^ and chickpeas^12,25^. In addition, a mutation at the Ala122 position of the *imi2* gene that was identified in wheat improved the resistance to IMI herbicides^12^.

The effectiveness of an herbicide depends on the retention of the product on the leaf^23^, the foliar absorption of the active ingredient and finally its translocation to the site of action^21,22^. Herbicide foliar retention is influenced by leaf morphological characteristics^36^, and it is not a major mechanism that confers herbicide resistance^23,37^. Our results suggest that the leaf morphology of wheat cultivars is not related to greater or lesser herbicide retention, as was demonstrated in other imazamox-resistant wheat cultivars^9,20,27^.

Imazamox is absorbed and translocated very quickly. However, given that resistance to ALS inhibitor herbicides is generally associated with mutations in the ALS gene^9–11^, the absorption and translocation are not usually studied, and information on these mechanisms in Clearfield® crops is scarce. We recorded high absorption rates between the S- and R-cultivars, confirming that this mechanism is not involved in the resistance of the latter. By contrast, the ^14^C-imazamox translocation results suggested that this parameter could play an important role in the resistance of the Rafalín and Antoñín cultivars because they retained most of the herbicide in the treated leaf. However, the translocation differences were a physiological and metabolic response to the different sensitivities to imazamox between the S- and R-cultivars, because this differential translocation was neutralized with malathion. The lowest translocation observed in the R-cultivars can be explained by the fact that these cultivars metabolized imazamox, and the identified metabolites (imazamox-OH and imazamox-glucose) have limited mobility^38^. Therefore, the observations made during the absorption and translocation assays in the wheat R-cultivars not only showed the translocated ^14^C-imazamox but also these ^14^C-metabolites.

Clearfield® crops generally have an enhanced ability to metabolize IMI herbicides before they reach the target site^39^. Imazamox metabolism was documented for the cultivar Clearfield® Pantera^20^, and although there are no studies in *Tritordeum*, it is evident that the R-cultivars (Rafalín and Antoñín) acquired the ability to metabolize IMI herbicides from their R counterparts. The mechanism by which Clearfield® crops gain tolerance to IMI herbicides has not yet been fully characterized^40^. In our study, malathion applications confirmed that Cyt-P450 enzymes play an important role in the imazamox detoxification of the R-cultivars into compounds that are harmless (imazamox-OH and imazamox-glucose) to the plants^22^. These enzymes are mediators of herbicide degradation that are involved in multiple herbicide resistance^11,41,42^. Cyt-P450 enzymes are responsible for the hydroxylation of a methyl group on the imazamox molecule studies^9,20^, followed by a glucosyl transferase catalysis producing a glucose conjugation^42^. This reaction occurs rapidly and is not reversible^41^. In addition, IMI herbicide metabolism not only has very little effect on the ALS, but the herbicide is also poorly translocated^39^. However, it can not be attributed that the metabolism of imazamox was due solely to Cyt-P450 genes, because other secondary metabolism pathways can be involved in resistance to ALS-inhibiting herbicides, resulting in an accumulation of different non-target-site-resistance genes, each of them conferring a moderate level of resistance^43^.

## Conclusions

The differential response to imazamox between R- and S-cultivars was primarily due to the number of resistance genes that carry each genotype (at target-site level), but it must also be attributed to the enhanced imazamox metabolism into non-toxic compounds (imazamox-OH and imazamox-glucose), which is mediated by the Cyt-P450 (non-target-site genes) and is responsible for altering the translocation patterns. These mechanisms confer high resistance to IMI herbicides in the cultivars Rafalín (*T. aestivum*) and Antoñín (*T. durum*), allowing them to survive at higher doses than the recommended field dose of imazamox (40 g ai ha^−1^), being a great advantage for farmers in terms of weed management.

## Material and Methods

### Plant material

Two wheat cultivars (*T. aestivum* and *T. durum*) were used. For *T. aestivum* the cultivar Rafalín resistant to imazamox (R), and the cultivar Gazul as the susceptible one (S) were used. For *T. durum*, the R- and S-cultivars were Antoñín and Simeto, respectively. The cultivar Rafalín comes from the crossbreeding of Pantera Clearfield® (R) x Gazul (S) cultivars. Pantera was previously characterized by this research group^20^, and it has two mutations (*imi1* and *imi2*) that confer resistant to imazamox. The resistant biotype of *T. durum* (Antoñín) comes from the crossing of Simeto (S) x *Tritordeum*, which presents the mutation *imi2* ^15^. The *Tritordeum*, likewise, comes from the crossing of *Triticum turgidum* x *Hordeum chilense*. The obtaining of the Antoñín cultivar and *Tritordeum* has been improving thanks to the work of the Plant Breeding group of Dr. Antonio P. Martín from the Institute for Sustainable Agriculture, Spanish National Research Council, Cordoba, Spain (IAS-CSIC).

The R-cultivars were selected survived a screening at the dose of 40 g ai ha^−1^ of imazamox (Pulsar® 40, imazamox 4%), and because they conserved the traits of their susceptible parents, Gazul and Simeto.

### Growth conditions

Seeds were sown in Petri dishes with two layers of filter paper moistened with distilled water. They were kept at 4 °C in the dark during 48 h. After this period, the seeds were transferred to a growth chamber until germination, with a temperature regime of 27/14 °C day/night with a photoperiod of 14/10 h, respectively. The seedlings were placed in pots (1 L) containing a mixture of peat and sand (1:1) as substrate, and taken to the greenhouse, where the plants grew at 25–28/12–14 °C day/night with 16 h of photoperiod. The natural light was supplemented by 900 µmol m^−2^ s^−1^ photosynthetic photon flux density delivered by incandescent and fluorescent lights. Once the plants reached a growth state corresponding to 3–4 true leaves, the treatments were performed.

### Foliar herbicide retention

Foliar herbicide retention assays were performed following the method adapted by Jiménez *et al*.^27^. Plants were treated with a solution containing 40 g ai ha^−1^ of imazamox + 1.25 L ha^−1^ of adjuvant Dash (34.5% w/v methyl oleate/methyl palmitate) + 100 mg L^−1^ Na-fluorescein in the same treatment chamber used in dose-response assays. Na-fluorescein was used as a labeling reagent to determine the amount of herbicide solution retained. Once the herbicide solution from the leaf (20–25 min) was dry, the plants were cut at ground level and washed individually in Erlenmeyer’s containing 50 mL of NaOH 5 mM shaking them vigorously for 30 seconds. The washing solution was recovered in glass flasks and the fluorescein absorbance was immediately measured at 490_exc_/510_em_ nm (Hitachi F-2500 spectrofluorimeter). The cut tissues were packed in cellulose envelopes and dried in an oven at 80 °C for 72 h. Ten plants of each cultivar were used in a completely random design. Retention was expressed as µL of imazamox solution per g of dry matter.

### Imazamox dose-response

Wheat plants were treated with imazamox at the following doses: 0, 2, 4, 8, 12, 16, 20 and 40 g ai ha^−1^ for S-cultivars, and 0, 40, 80, 120, 160 and 240 g ai ha^−1^ for R-cultivars. Dash adjuvant was added at dose of 1.25 L ha^−1^ in all treatments. Herbicide applications were conducted using a treatment chamber (Devries Manufacturing, Hollandale, MN, USA) equipped with an 8002EVS flat fan nozzle (TeeJet, Spraying System Spain, S.L., Madrid, Spain) calibrated at 200 kPa and 250 L ha^−1^ of application volume. The experiment was repeated twice in a completely randomized design with 10 replicates per dose, evaluating the fresh weight reduction of the plants at 30 days after application (DAA). Data were expressed as percentage fresh weight reduction with respect to the untreated control plants.

### ALS enzyme activity

ALS activity was determined following the methodology used by Hatami *et al*.^44^ with slight modifications. Samples of three grams of leaf tissue were taken and immediately frozen in liquid N_2_. Then, the samples were macerated in a mortar using 5 mg of polyvinylpyrrolidone (PVPP). An extraction buffer composed of 1 M K-phosphate buffer solution (pH 7.5), 10 mM sodium pyruvate, 5 mM MgCl_2_, 50 mM thiamine pyrophosphate, 100 μM flavin adenine dinucleotide (FAD), 12 mM dithiothreitol and glycerol (1:9 v/v) was added. The solution was agitated for 10 min at 4 °C. The homogenate was filtered through four layers of cheesecloth and centrifuged (20,000 rpm for 20 min). The supernatant containing a crude ALS enzyme extract was immediately used for the enzyme assays. To assay the ALS activity, 90 µL of enzyme extract was added to 110 µL of freshly prepared assay buffer (0.08 M K-phosphate buffer solution (pH 7.5), 0.5 M sodium pyruvate, 0.1 M MgCl_2_, 0.5 mM thiamine pyrophosphate, and 1 µM FAD). Then, increasing concentrations (0, 0.1, 1, 5, 10, 50, 100 and 1000 µM) of ALS inhibiting herbicides were added. The herbicides of technical grade were imazamox, imazapyr, imazaquin, tribenuron methyl, bispyribac sodium, flucarbazone and florasulam. Standard compounds used with 96.5–98% purity were provided by Sigma-Aldrich, Spain. The mixture was incubated for 60 min at 37 °C. The reaction was stopped after the addition of 50 µL of H_2_SO_4_ and incubated at 60 °C for 15 min to decarboxylate acetolactate to acetoin. Finally, 250 µL of a freshly prepared solution of creatine in water (5 g L^−1^) and 250 µL of a solution of naphthol in sodium hydroxide (50 g L^−1^ NaOH 5 M) was added. It was again incubated at 60 °C for 15 min to facilitate decarboxylation of acetolactate to acetoin. Absorbance of acetoin was measured with a spectrophotometer (Beckman DU-640, Fullerton, CA, USA) at A520 nm. The total content of ALS in the raw extract was measured using the colorimetric method using the commercial kit-protocol No. P5656 (Sigma-Aldrich, Madrid, Spain) following the manufacturer´s instructions at 595 nm. The background was subtracted using control tubes. Three replicates per cultivar were made, each with extract from the mixture of the three plants.

### ALS sequencing

Young leaf samples (±100 mg) from four wheat cultivars were taken and stored at −20 °C, until use. For DNA extraction, the Speed tools kit DNA Extraction Kit Cat Plant (Biotools B & M Labs. S.A) were used. The primer pair AHAS21Fwd/AHAS26Rev, designed by Pozniak *et al*.^16^ to amplify a 617 bp-length fragment was observed. A polymerase chain reaction (PCR) reaction was set up with Certamp complex enzyme mix (Biotools B&M Labs, Madrid, Spain) following the manufacturer’s instructions. PCR products (5 µL) were digested with the restriction enzyme *Msp I* (Invitrogen, CA, USA) to try to identify the three expected ALS alleles (from genomes A, B or D) of the catalytic subunit present in wheat varieties^16^. Both PCR and digestion products were resolved on 1% agarose gels and viewed under UV light. Ten PCR products of each allele and each cultivar were sequenced by Sanger technology. The assembly of the sequences was carried out by SeqMan Pro (Version 11, DNASTAR; Madison, WI, USA) and Geneious (Version 8.1.8, Biomatters Ltd, and Auckland, New Zealand) software’s. ALS sequences of the wheat accessions *imi1*-AY210407, *imi2*-AY210408 and *imi3*-AY273827 from GenBank, were included in the alignment.

### ^14^C-imazamox absorption-translocation

Wheat plants were treated with an herbicide solution prepared with commercial product mixed with ^14^C-imazamox. The final concentration corresponded to 40 g ai ha^−1^ of imazamox + 1.25 L ha^−1^ of Dash into 250 L ha^−1^ with a specific activity of 834 kBq μL^−1^. A drop (1 μL/plant) of this solution was applied to the surface of the second expanded leaf using a micropipette (Lab Mate HTL). The treated plants were carefully removed from the pot and washed at 3, 6, 12, 24, 48 and 96 HAA. ^14^C-imazamox unabsorbed from a treated leaf was washed with 3 mL of water-acetone (9:1 v/v) solution. Plants were separated into treated leaf, remainder of plant, and roots. The rinsing solution was mixed with 2 mL of scintillation fluid (Ultima GoldTM; Perkin-Elmer, Packard Bioscience BV) and analyzed by the LSS detector (scintillation counter, Beckman LS 6500). Samples of the plants were stored individually in combustion cones (Combuste-Cone, Flexible: Perkin-Elmer, Packard Bioscience BV), dried in an oven at 60 °C for 72 h, and then combusted using a biological oxidizer (Packard Tri Carb 307, Packard Instruments, Meriden, CT, USA). The CO_2_ produced in the combustion was retained in 18 mL of a mixture of Carbo-Sorb E and Permafluor (1:1 v/v) (Perkin-Elmer, BV Bioscience Packard) in scintillation vials. Radioactivity was quantified by LSS, and the percentage of absorbed herbicide was expressed as [KBq tissue oxidized by combustion / (KBq oxidized by combusting + KBq tissue obtained from washing)] × 100. Five plants of each cultivar were used in a completely random design.

Simultaneously, whole plants treated with ^14^C-imazamox and rinsed with water: acetone (9:1 v/v), were fixed on filter paper (25 × 12.5 cm) and dried at room temperature. Finally, they were placed for 4 h on a phosphor film to visualize the ^14^C-imazamox by a phosphor imager (Cyclone, Perkin-Elmer, and Packard Bioscience BV). Three plants of each cultivar were used at each evaluation time.

### Imazamox metabolism

The methodology described by Rojano-Delgado *et al*.^45^ was followed. Ten plants of each wheat cultivar were treated with imazamox at 40 g ai ha^−1^ (field dose) as in the dose-response assays. Jointly, a group of 10 plants of each cultivar were treated with Malathion (1000 g ai ha^−1^) 1 h before imazamox application, to evaluate if the Cyt-P450 was involved in the imazamox metabolism. A group of plants was remained as control. Those plants treated with herbicide and the controls were cut at 96 HAA and were washed with 60 mL of distilled water to remove the imazamox and soil residues on the leaf surface and finally stored at −40 °C until use. The samples were macerated in a porcelain mortar to a fine powder using liquid nitrogen. Next, 500 mg of each sample was mixed with 10 mL of methanol:water (9:1 v/v), and the metabolites were extracted using ultrasound at 70-W ultrasonication power for 10 min (duty cycle 0.7 s s^−1^). Supernatant was separated by centrifugation (15 min at 15000 rpm), and evaporated to dryness under an airstream. The solid residue of this fraction was reconstituted in 500 µL of methanol:water (9:1 v/v), and filtered through a nylon filter syringe (45 μm pore size and 13 mm i.d.; Millipore, Ireland) before chromatographic analysis.

For the determination of imazamox and its metabolites in extracts from plants, a liquid chromatography-diode array detector was employed. A hydrophilic interaction liquid chromatography column C18 (20 cm × 4.6 cm, 3 μm particle size) was used for the separation of the target compounds. Fifty µL of the reconstituted phase was injected into the liquid chromatography with 1% acetic acid solution as mobile phase A, and 100% methanol as mobile phase B. The elution program started with 5% mobile phase B and followed the linear gradient: step 1: 5 to 20% methanol for 10 min; step 2: 20 to 80% methanol for 10 min; step 3: 80 to 100% methanol for 5 min; and step 4: 100 to 5% methanol for 10 min. The constant flow rate and column temperature were 1 mL min^−1^ at 40 °C. Chromatographic grade and liquid chromatography–mass spectrometer grade solvents were used for liquid chromatography-diode array detector and liquid chromatography-time-of-flight/mass spectrometer analysis, respectively.

The analyses were performed in an Agilent 1200 Series LC system interfaced to an Agilent 6540 UHD Accurate-Mass liquid chromatography–time-of-flight/mass spectrometer detector (Palo Alto, USA), equipped with an Agilent Jet Stream Technology electrospray ion source operating in the positive ionization mode. The separation conditions were identical to those for the liquid chromatography–diode array detector determination, except for the use of the respective liquid chromatography–time-of-flight/mass spectrometer grade solvents.

After analysis, imazamox and metabolites were determined by the liquid chromatography-diode array detector analysis (measurement wavelength, 240 nm). A 15 Gold HPLC System from Beckman Coulter (Fullerton, USA) equipped with a 26 System Gold Diode Array detector (wavelength range 190–600 nm) was used in this case. Chromatographic peaks were assigned according to retention times using as a reference the imazamox peak identified by spiking extracts with the commercial standard. Quantification of imazamox metabolites was based on the calibration model for imazamox, and the results were expressed as μg of analytic g^−1^ fresh weight. Three replications per sample were analyzed.

### Statistical analysis

The percentage data of fresh weight reduction and ALS enzyme activity were submitted to a non-linear regression analysis. The dose of imazamox needed to reduce the weight of a population (GR_50_) and to inhibit ALS activity (I_50_) by 50% was calculated. The *drc* statistical package in the program R version 3.2.5 was used to conduct the following log-logistic model of four parameters^46^: *Y* = *c* + {(*d* − *c*)/[1 + (x/*g*) b]}, where *Y* is the percentage of fresh weight reduction with respect to the control, *c* and *d* are coefficients corresponding to the upper and lower asymptotic limits, *b* is the Hill slope, *g* is the imazamox dose (GR_50_, or I_50_) at the mean point of inflexion between the upper and lower asymptote and x (independent variable) corresponds to the glyphosate dose. The data were plotted using SigmaPlot 11.0 (Systat Software, Inc., USA). Resistance factor was calculated as: RF = GR_50_ or I_50_ (R)/GR_50_ or I_50_ (S).

The data obtained in the spray retention, ^14^C-imazamox absorption and translocation, and imazamox metabolism were subjected to ANOVA. For each analysis, assumptions such as equality of variance and normal distribution were evaluated. When required, the Tukey HSD test at 5% probability was used to separate means. A statistical analysis was performed using Statistix software (version 9.0) from Analytical Software (USA).
